# A brain and a head for a different habitat: Size variation in four morphs of Arctic charr (*Salvelinus alpinus* (L.)) in a deep oligotrophic lake

**DOI:** 10.1002/ece3.6771

**Published:** 2020-09-25

**Authors:** Ana‐Maria Peris Tamayo, Olivier Devineau, Kim Præbel, Kimmo K. Kahilainen, Kjartan Østbye

**Affiliations:** ^1^ Faculty of Applied Ecology, Agricultural Sciences and Biotechnology Inland Norway University of Applied Sciences Koppang Norway; ^2^ Norwegian College of Fishery Science, Faculty of Biosciences, Fisheries and Economics UiT—The Arctic University of Norway Tromsø Norway; ^3^ Lammi Biological Station University of Helsinki Lammi Finland; ^4^ Centre for Ecological and Evolutionary Synthesis (CEES), Department of Biosciences University of Oslo Oslo Norway

**Keywords:** adaptive radiation, ecological speciation, evolution, phenotypic plasticity, polymorphism, species complex

## Abstract

Adaptive radiation is the diversification of species to different ecological niches and has repeatedly occurred in different salmonid fish of postglacial lakes. In Lake Tinnsjøen, one of the largest and deepest lakes in Norway, the salmonid fish, Arctic charr (*Salvelinus alpinus* (L.)), has likely radiated within 9,700 years after deglaciation into ecologically and genetically segregated Piscivore, Planktivore, Dwarf, and Abyssal morphs in the pelagial, littoral, shallow‐moderate profundal, and deep‐profundal habitats. We compared trait variation in the size of the head, the eye and olfactory organs, as well as the volumes of five brain regions of these four Arctic charr morphs. We hypothesised that specific habitat characteristics have promoted divergent body, head, and brain sizes related to utilized depth differing in environmental constraints (e.g., light, oxygen, pressure, temperature, and food quality). The most important ecomorphological variables differentiating morphs were eye area, habitat, and number of lamellae. The Abyssal morph living in the deepest areas of the lake had the smallest brain region volumes, head, and eye size. Comparing the olfactory bulb with the optic tectum in size, it was larger in the Abyssal morph than in the Piscivore morph. The Piscivore and Planktivore morphs that use more illuminated habitats have the largest optic tectum volume, followed by the Dwarf. The observed differences in body size and sensory capacities in terms of vision and olfaction in shallow and deepwater morphs likely relates to foraging and mating habitats in Lake Tinnsjøen. Further seasonal and experimental studies of brain volume in polymorphic species are needed to test the role of plasticity and adaptive evolution behind the observed differences.

## Graphical abstract

In Lake Tinnsjøen, one of the largest and deepest lakes in Norway, the salmonid fish, Arctic charr (*Salvelinus alpinus* (L.)), have likely radiated within 9,700 years after deglaciation into ecologically and genetically segregated Piscivore, Planktivore, Dwarf, and Abyssal morphs in the pelagial, littoral, shallow‐moderate profundal, and deep‐profundal habitats. The observed differences in body size and sensory capacities in terms of vision in shallow and deepwater morphs likely relate to foraging and mating habitats in Lake Tinnsjøen.

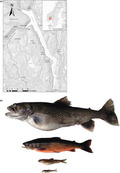

## 1 INTRODUCTION

Resource polymorphism occurs in a range of different species with intraspecific morphs, originating from phenotypic plasticity, adaptive evolution, or both of these processes, thorough use of different habitat and diet as a response to ecological opportunity within available niches (Skúlason et al., 2019). Resource polymorphism is specially common in the salmonid genera of *Salvelinus* and *Coregonus*, which often show phenotypical divergence in pelagic and benthic niches in postglacial lakes (e.g., Guiguer, Reist, Power, & Babaluk, 2002; Kahilainen, Malinen, Tuomaala, & Lehtonen, 2004; Muir, Hansen, Bronte, & Krueger, 2016; Smalås, Amundsen, & Knudsen, 2013). The most common occurrence of polymorphism consists of two sympatric morphs inhabiting well‐lit littoral and pelagic habitats, whereas some large and deep lakes can have more pronounced resource polymorphism with 3–8 morphs, including deepwater profundal morphs (Doenz, Krähenbühl, Walker, Seehausen, & Brodersen, 2019; Kahilainen & Østbye, 2006; Markevich, Esin, & Anisimova, 2018; Power, O'Connell, & Dempson, 2005; Skoglund, Siwertsson, Amundsen, & Knudsen, 2015). Moreover, growth rates, spawning habitat and time, age, size, and colour patterns at sexual maturity can also differ amongst sympatric morphs in these genera (Kahilainen & Østbye, 2006; Sandlund et al., 1992; Walker, Greer, & Gardner, 1988). While such morphological and life‐history differences of *Salvelinus* and *Coregonus* are increasingly well documented throughout their distribution range, there are no previous studies on putative divergence in sensory capacities in terms of brain structure.

Brain morphology varies across vertebrate taxa, with the development of different structures depending on factors such as environmental conditions (e.g., oxygen and pressure), predation, habitat, diet, and social interactions (e.g., Crispo & Chapman, 2010; Day, Westcott, & Olster, 2005; Edmunds, Laberge, & McCann, 2016; Harvey, Clutton‐Brock, & Mace, 1980; Yopak, Lisney, Collin, & Montgomery, 2007). Occupying different environments requires different traits, which can varies with depth (Caves, Sutton, & Johnsen, 2017). For instance, adaptations to a deepwater habitat in freshwater and marine systems can involve changes in morphology (e.g., eye size), lowered rates of metabolism, variation in the oxygen transport system, and fatty acid composition (e.g., Evans, Præbel, Peruzzi, Amundsen, & Bernatchez, 2014; Kahilainen & Østbye, 2006; Radnaeva et al., 2017; Seibel & Drazen, 2007). Brain morphology can also be affected by depth, turbidity, and feeding type, such as the development of a larger optic tectum and larger eyes in fish feeding on active prey in well‐illuminated habitats and low turbidity (Huber, van Staaden, Kaufman, & Liem, 1997). Natural selection may act on the brain, targeting morphology, and adaptive function of different regions under divergent selection, also being active below the species level such as morphs using different niches (Gonda, Herczeg, & Merilä, 2013; Merilä & Crnokrak, 2001).

Regarding metabolism, energetic costs can constrain the development of the brain size as it is one of the most energetically expensive organs (Kotrschal et al., 2013; Laughlin, van Steveninck, & Anderson, 1998). The increase in size and complexity of the brain can be a trade‐off between selection for cognitive benefits and the cost of production and maintenance of the brain (Gonda et al., 2013; Kotrschal et al., 2014). The brain, as the controller of behaviour and eco‐physiological functions, can be under developmental canalization (i.e., the ability of a genotype to produce one or a few targeted phenotypes in different environments, presenting a lack of plasticity) or under phenotypic plasticity (Ghalambor, McKay, Carroll, & Reznick, 2007; Gottlieb, 1991). Since phenotypic plasticity may be either adaptive, or nonadaptive, not all plasticity will necessarily provide a fitness advantage (Ghalambor et al., 2007).

Brain structure of fish is similar to other vertebrates (Kotrschal, Van Staaden, & Huber, 1998). In fish, olfactory organs are composed by lamellae and are attached to the olfactory nerves. These nerves are connected to the olfactory bulb, which processes information about odours, and it is thus involved in social communication, feeding and mating behaviour, and predator recognition (Chivers & Smith, 1993; Dulka, 1993; Hara, Sveinsson, Evans, & Klaprat, 1993; Landry, Garant, Duchesne, & Bernatchez, 2001; Milinski et al., 2005). An enlargement of the olfactory bulb can be found in fish that live in environments with high predation risk (Gonda, Valimaki, Herczeg, & Merila, 2012). The telencephalon and hypothalamus are related to more complex activities such as learning, memory and social tasks (Demski, 1983; Kotrschal et al., 1998). For instance, fishes living in structured environments show a larger telencephalon (Huber et al., 1997). The hypothalamus is also involved in regulating reproductive and feeding behaviour (Kulczykowska & Vázquez, 2010; White & Fernald, 1993). Gonda et al., (2012) found a reduction of the hypothalamus in the presence of predation in nine‐spined sticklebacks that were less aggressive and took less risks to feed than in absence of predators (Herczeg & Välimäki, 2011).

Eyes and the optic tectum are involved in vision, and both of these structures are used as an indicator of visual capabilities and importance (Huber et al., 1997; Lisney, Bennett, & Collin, 2007). The cerebellum is in charge of several tasks such as motor coordination, proprioception (i.e., movement and balance), and eye movement (Demski, 1983). In addition, habitat complexity can also influence the brain regions, increasing the cerebellum and telencephalon size, and decreasing the olfactory bulb (Pollen et al., 2007). Social environment seems to affect the brain as well, increasing the optic tectum size and decreasing the olfactory bulb when fish live in groups (Gonda, Herczeg, & Merilä, 2009). Many of the above brain volume studies have been conducted with shallow water species in lakes with well‐illuminated habitats lacking strong vertical gradients of light, temperature, pressure, and prey availability. Such conditions prevail in many deep and oligotrophic lakes inhabited by polymorphic fish, but we do not know the potential effects of such depth gradients and habitat selection on corresponding brain morphology.

**FIGURE 1:**
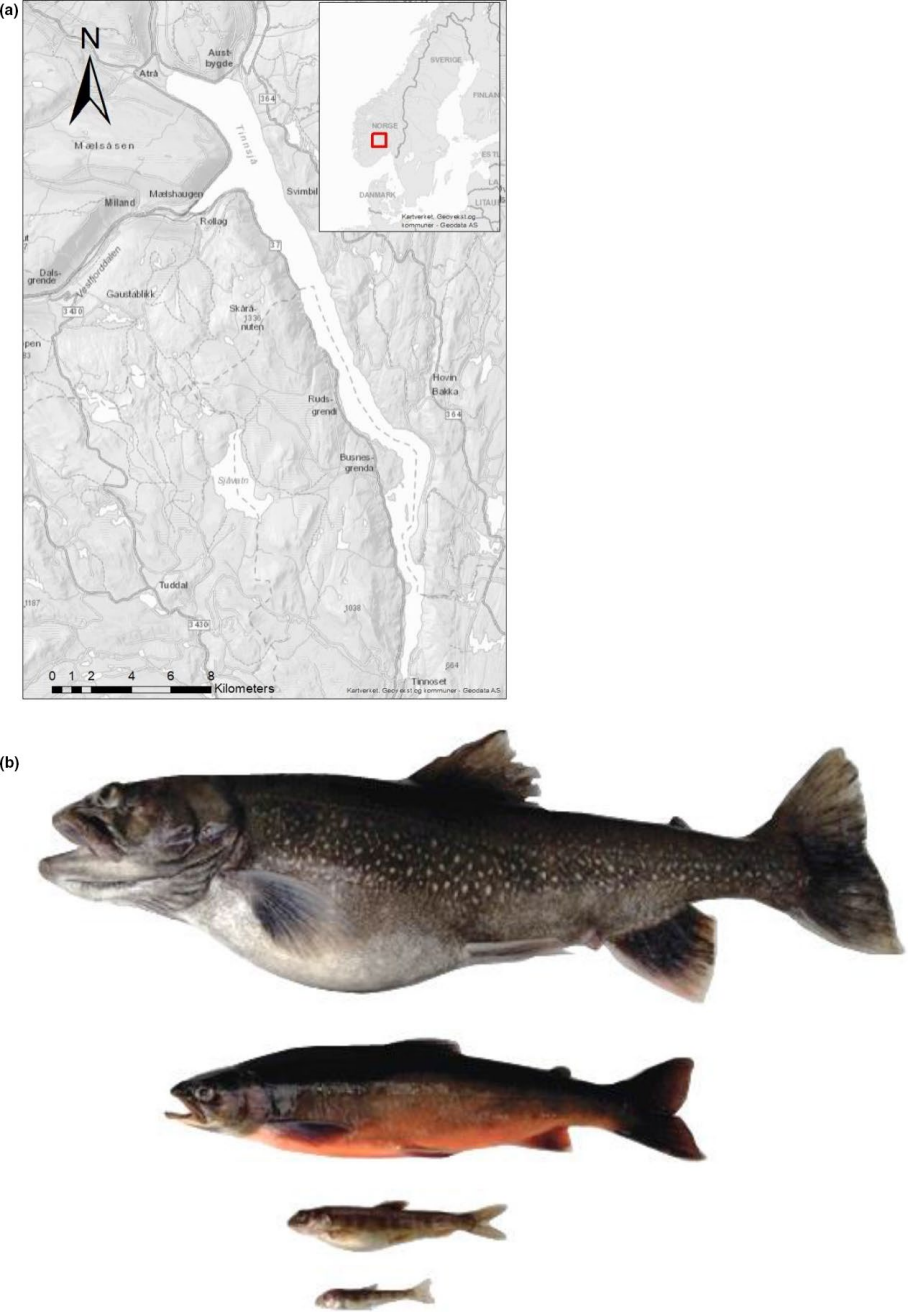
(a) Study area of Lake Tinnsjøen, Norway. Exact sampling positions are not reported until more information is available about the population status of the new Abyssal morph. 1:125,000 using ArcGIS (ESRI 2015). (b) Sexually mature fish from each of the four Arctic charr morphs in Lake Tinnsjøen. From top to bottom: Piscivore (greyish colour and inflated swim bladder; Body length: 217.38 ± 92.96, mean ± *SD*), Planktivore (male in spawning dress with orange belly; 177.27 ± 63.35), Dwarf (brown with inflated swim bladder; 113.82 ± 21.50), and Abyssal (sunken eyes; 78.58 ± 10.66). (Photo © K. Østbye)

In the deep oligotrophic Lake Tinnsjøen, in southern Norway, four Arctic charr morphs coexist along steep depth gradients (Figure 1). This lake contains two profundal morphs, the Dwarf and Piscivore morphs, one Planktivore, a habitat generalist morph, and one deep‐profundal benthivore morph, the Abyssal morph (Østbye et al., 2020). All these morphs presented differences in body size and coloration (Østbye et al., 2020). The Piscivore is the largest morph having a large, robust head and elongated black/grey body, showing a piscivorous behaviour, feeding on other fish, while the Dwarf is a small‐bodied morph with a pale brown coloration often with parr marks, feeding on macrobenthos and zooplankton. The Planktivore is a moderately sized morph with a darkish coloration on the upper part of the body with silvery sides, and feeds on zooplankton. Finally, the minute Abyssal morph is a tiny fish with a pale bluish‐whitish body colour, light purple coloration on parts of its head, and it feeds on the soft‐profundal‐bottom benthic invertebrates. These striking phenotypic differences coupled with largely contrasting environmental conditions in their habitats, strongly imply putative sensory divergence in different lake habitats.

In this study, we tested how different habitat use along a depth gradient may correspond to head morphology and brain volume in the four Arctic charr morphs in Lake Tinnsjøen. First, we aimed to detect clustering of four morphs based on combined differences in ecomorphology, population genetics, life‐history traits, and brain volume, using recursive partitioning methods. Secondly, we aimed to compare brain variation and sensory traits among the four morphs. We hypothesised that the morphs (i.e., Planktivore, Piscivore, and Dwarf) living in habitats with more light radiation will have a larger optic tectum than the Abyssal morph. We hypothesised that the Abyssal morph will have developed a better smell perception due to lack of light in the deep‐profundal habitat (Yopak et al., 2019), showing abundant lamellae, larger surface of the olfactory rosette, and a developed olfactory bulb. Finally, we hypothesised that the Abyssal morph will have the smallest brain regions due to prey resource limitation in their habitat.

## 2 METHODS

### 2.1 Study area

Lake Tinnsjøen (60°38ʹ15.6″N, 11°07ʹ15.2″E, elevation 191 m.a.s.l.) in Telemark county, southern Norway (Figure 1) is one of the largest lakes in Norway (51.4 km^2^), and one of the deepest in Europe (max. depth 460 m). It is an oligotrophic lake harbouring Arctic charr, brown trout (*Salmo trutta*), a small population of perch (*Perca fluviatilis*) and the recently introduced minnow (*Phoxinus phoxinus*). According to Boehrer, Golmen, Løvik, Rahn, and Klaveness (2013), oxygen concentration in June 2006 ranged from 11.5 to 12.0 mg/L, dissolved oxygen from 90% to 85% at 0–460 m depth, and temperature ranged from 4.0 to 3.3°C at 50–460 m. A river connected the lake to the sea during the most recent postglacial period (9,700 years to present; Bergstrøm, 1999), which suggests that the fish fauna colonized the lake naturally after deglaciation via this river.

### 2.2 Fish collection

We sampled fish using gillnets, traps and baited anchored longlines in August–October 2013 (Østbye et al., 2020). We sampled in four habitats: (i) the pelagial (setting gillnets positioned more than 50 m from shore and 20–30 m depth in midwater using a 12‐panel multimesh Nordic series with mesh sizes in this order of 43, 19.5, 10.0, 55.0, 12.5, 24, 15.5, 35.0, 29.0, 6.3, 5.0 and 10.0 mm and Jensen floating series with mesh size of 13.5, 16.5, 19.5, 22.5, 26.0, 29.0, 35.0, 39.0, 45.0 and 52.0 mm), (ii) the littoral (gillnets within 20 m from the shore using Nordic and Jensen littoral net series), (iii) the shallow‐moderate profundal (Jensen littoral net series, traps, and hook‐line between 20 and 150 m depth), and (iv) the deep profundal (setting traps >150 m depth and >100 m from the shoreline using longlines of 220 m long and 3 to 4 mm line with 180 hooks; see more detailed information in Østbye et al., 2020). In the field, we assigned each individual to one of the four morphs (called field‐assigned morphs: FA morphs) based on differences in body and head appearance and coloration. We also measured body length and determined the sex and maturation stage visually (i.e., mature if the gonads covered more than half of the body cavity length; immature otherwise). We euthanized the fish with an overdose of benzocaine, and we preserved the heads in formalin (10% unbuffered).

### 2.3 Genetic analyses

We had 72 individuals with both genetic and morphological data for each individual (field assigned morphs: Planktivore (*n* = 25), Piscivore (*n* = 13), Dwarf (*n* = 22), and Abyssal (*n* = 12); Figure 1). In the deep‐profundal habitat, only the Abyssal morph was caught. The Dwarf and Piscivore morphs were caught in the shallow‐moderate profundal habitat. The Planktivore morph was caught in the pelagial (*n* = 8), littoral (*n* = 6), and shallow‐moderate profundal habitat (*n* = 11). We used 10 microsatellite markers to classify the fish into genetic clusters (*K*; see Østbye et al., 2020). Herein, we used allele frequencies to identify the genetic clusters of Arctic charr (genetic assigned morphs, GA‐morphs) with the software STRUCTURE (Pritchard, Stephens, & Donnelly, 2000). We included a predictor variable to test whether pure and hybrid individuals differed based on *q*‐value (i.e., using admixture proportions of individuals; Bhat et al., 2014), considering a threshold value of *q* > 0.7 for genetically pure individuals (i.e., belonging to a unique cluster), and *q* < 0.7 for hybrids (Anderson & Thompson, 2002; Harrison, 1993). In Lake Tinnsjøen, 48 fish were genetically pure (genetic cluster 1 was the Planktivore morph (*n* = 12), genetic cluster 2 was the Piscivore morph (*n* = 15), genetic cluster 3 was the Dwarf morph (*n* = 13), and genetic cluster 4 was the Abyssal morph (*n* = 8)) and 24 hybrids were also identified (Østbye et al., 2020). All the analyses below are based on the genetic classification (GA‐morphs).

### 2.4 Head morphometrics

**FIGURE 2:**
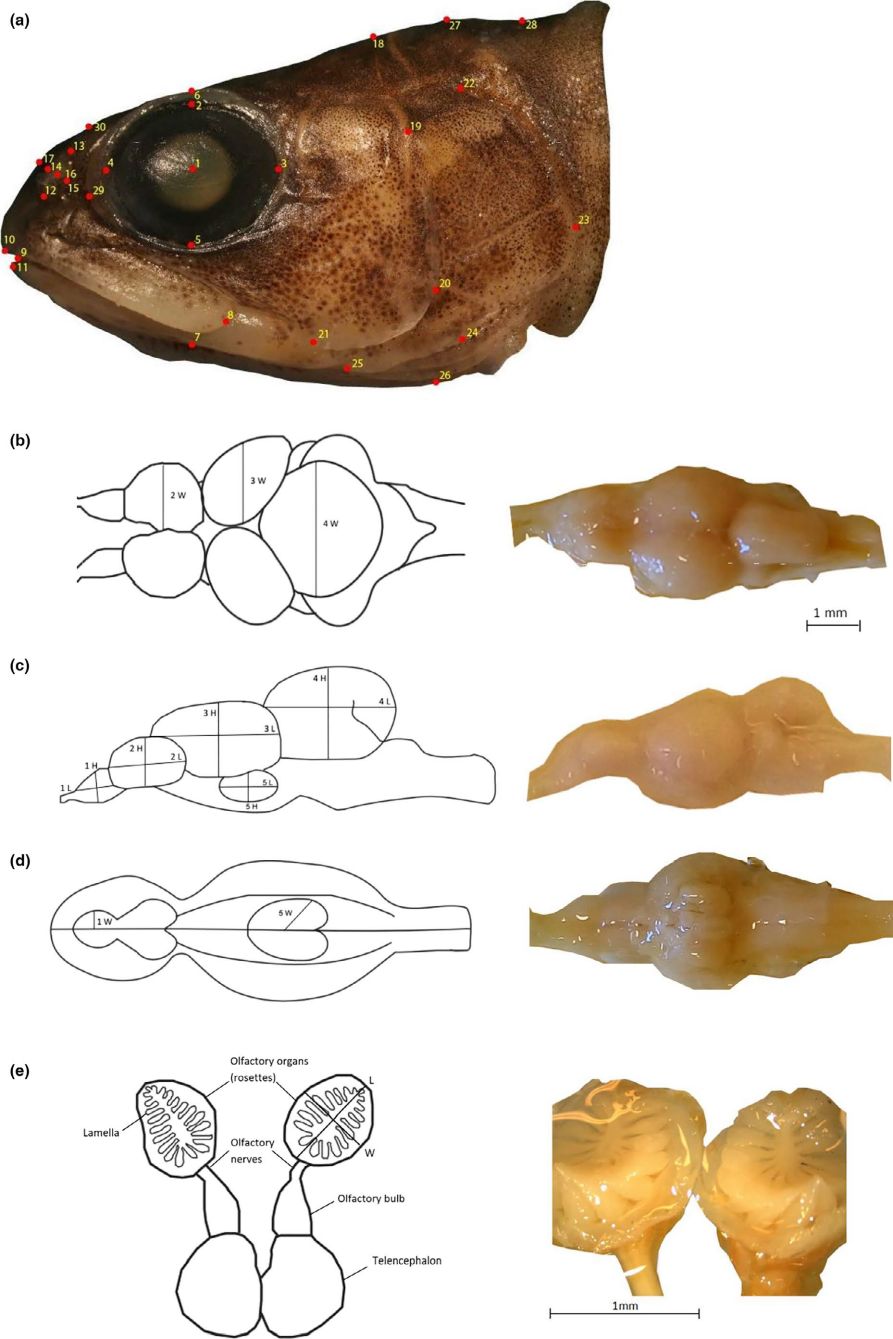
(a) Anatomical landmarks (30 points) used to measure the head shape of Arctic charr. Photo from male Dwarf morph, which was a genetically pure individual (*q* = 0.85). Landmarks used for head shape analysis: 1. Central point of the eye, 2. Dorsal extreme of bony orbit of the eye, 3. Posterior extreme of bony orbit of the eye, 4. Anterior extreme of bony orbit of the eye, 5. Ventral extreme of bony orbit of the eye, 6–7. Perpendicular line following landmarks 1, 2 and 5, 8. Posterior point of the upper jaw, 9. Central point of the closed mouth, 10. Anterior point of the upper jaw, 11. Anterior point of the lower jaw, 12. Ventral extreme of nostril, 13. Dorsal extreme of nostril, 14. Anterior point of nostril, 15. Posterior point of nostril, 16. Central point of nostril, 17. Perpendicular line following landmarks 14, 15 and 16, 18. Starting point of the line of preoperculum, 19. Upper point of the preoperculum, 20. Point of maximum curvature of the preoperculum, 21 Lower point of preoperculum, 22. Upper point of the operculum, 23. Posterior point of the bony operculum, 24. Point of curvature of the operculum, 25. Lower point of operculum, 26. Perpendicular line following landmark 20 to the bottom of the fish, 27. Middle point between landmarks 18 and 28, 28. Starting point of the line of operculum, 29. Socket of the eye, 30. Perpendicular line from landmark 29. (b) Dorsal, (c) lateral and (d) ventral view of the brain, illustrating the five brain regions studied (1: olfactory bulb, 2: telencephalon, 3: optic tectum, 4: cerebellum, 5: hypothalamus). For each brain region, the length (L), height (H), and width (W) were measured. (e) Olfactory lamellae of Arctic charr and illustration of olfactory organs with lamellae and olfactory nerves attached with the olfactory bulb, which is connected with the telencephalon

We photographed the left side of each fish using a digital camera (Canon EOS 350D), and we preprocessed the photographs with tpsUtil v.1.26 (Rohlf, 2004). We digitized a set of 30 common anatomical landmarks in tpsDIG2 v.2.22 (Rohlf, 2015) to capture head variation (Figure 2a), which we included for landmark‐based geometric morphometrics and statistical analyses. In addition, we measured the width (W) and height (H) of the eye in tpsDIG2 to calculate the eye area.

### 2.5 Age determination

We determined the age based on otoliths, which are more reliable than scales especially in Arctic charr (Christensen, 1964). We opened the skull dorsally under a microscope and removed the olfactory rosette and the brain from the olfactory bulb to the spinal cord to collect the otoliths. We used a microscope to count the rings of the dorsal part of otoliths for determining age. We then burned one otolith with a gas flame for ca. 5 s and broke it in half to count the rings from the lateral side under a microscope, as a further confirmation of age.

### 2.6 Neuroanatomy

Following Pollen et al. (2007), we measured five brain regions (Figure 2b–d): olfactory bulb, telencephalon, optic tectum, cerebellum, and hypothalamus. We measured the width (*W*) of each brain structure from the dorsal and ventral image of the brain, as well as the length (*L*) and height (*H*) from lateral views of the left hemisphere (Figure 2b–d). We used an ellipsoid model to estimate the volume (*V* = 1/6 *π*(*LWH*)) of each brain region (Huber et al., 1997).

### 2.7 Olfactory rosettes

We dissected the olfactory rosettes and the nasal organ and stored them in 70% ethanol (Figure 2e). We measured the width (*W*) and length (*L*) using a micrometer under the microscope in order to calculate the surface area of each olfactory rosette (*A* = 1/4*π*(*WL*)). We also counted the number of olfactory lamellae in each rosette.

### 2.8 Statistical analysis

#### 2.8.1 Quantification of diversity in the morphs

We conducted a principal component analyses (PCA) to evaluate the variation in head shape among morphs. We standardized for size with a Generalized Procrustes Analysis (Adams, Rohlf, & Slice, 2004; Zelditch, Swiderski, Sheets, & Fink, 2004). We then conducted a PCA using the package geomorph (Adams & Otárola‐Castillo, 2013). We calculated the centroid size (i.e., as a measure of size) for each individual to use in further analyses (i.e., analysis of variance (ANOVA), post hoc Tukey's HSD and random forest).

To account for allometric relationships, we used log‐log regression approach for each morphological measurement, using body length as predictor. We performed ANOVAs and post hoc Tukey's HSD for all the variables to know whether there were differences among the morphs. We used the residuals from the regressions for the ANOVAs, post hoc Tukey's HSD, and random forest analyses to account for size.

### 2.9 Morph prediction using random forest

Recursive partitioning methods are used in fields such as genetics, psychology, medicine, and epidemiology (Qi, Bar‐Joseph, & Klein‐Seetharaman, 2006; Segal, Barbour, & Grant, 2004; Shen, Ong, Li, Hui, & Wilder‐Smith, 2007; Ward, Pajevic, Dreyfuss, & Malley, 2006), but less in ecology (Cui et al., 2019; Cutler et al., 2007; Desantis, 2019; Kargar, Akhzari, & Saadatfar, 2019). To differentiate between morphs, including all the variables measured above, we used a random forest approach with 10‐fold cross‐validation and 5 repeats per fold. To predict the four GA‐morphs, we opted for a random forest approach rather than a regression because there was a higher number of parameters than observations (Strobl, Malley, & Tutz, 2009). We thus discuss the variable importance rather than parameters estimates below (Rossi, Amaddeo, Sandri, & Tansella, 2005). Compared to principal components or discriminant analyses, random forests are more flexible (Kuhn & Johnson, 2013; Zhang & Aires‐de‐Sousa, 2007; Zumel & Mount, 2014), are robust to overfitting, have internal cross‐validation, and often outperforms more classical approaches in terms of prediction accuracy (Johnston, Johnston, Kennedy, & Florence, 2008; Kuhn & Johnson, 2013; Palmer, O'Boyle, Glen, & Mitchell, 2007; Svetnik et al., 2003; Zhang & Aires‐de‐Sousa, 2007).

Random forest is an ensemble method, which builds many decision trees to obtain more accurate classifications (Cutler et al., 2007; Strobl et al., 2009). We generated 5,000 trees, with 3 variables considered for each split (Bischl et al., 2016), which was calculated as the square root of the number of predictors. To train the random forest model, we included the variables habitat (littoral, pelagial, shallow‐moderate profundal and deep‐profundal), genetic trait (pure/hybrid), sex (male/female), maturation (mature/immature), number of olfactory lamellae, area olfactory rosette, eye area, all volumes of the different brain regions (olfactory bulb, telencephalon, optic tectum, cerebellum, hypothalamus), head size (i.e., as the centroid size for each individual calculated as the average of *x* and *y* coordinates of all landmarks), and the age of fish. We then identified the most important variables to predict the four morphs. Note that we used the residuals of the variables obtained from the log‐log regressions to correct for size. We estimated the accuracy of the random forest. We assessed the relative contribution of variables to the classification with variable importance, ranking predictors by the mean minimal depth (Ishwaran, Kogalur, Chen, & Minn, 2011; Paluszynska, Biecek, & Jiang, 2019). We used accumulated local effects (ALE) plots to visualise the variables influence in the prediction of the model (Friedman, 2001). When the ALE values are positive, there is a higher probability to belong to a specific class. We only report the ALE plots for the most important variables. We used R packages ranger (Wright, Wager, & Probst, 2019) for the analysis, iml (Molnar, 2018) for the ALE plots, and randomForestExplainer (Paluszynska et al., 2019) for the variable importance plot. All statistical analyses were performed in R version 3.6.1 (R Core Team, 2019).

## 3 RESULTS

### 3.1 Quantification of diversity in the morphs—head shape

**FIGURE 3:**
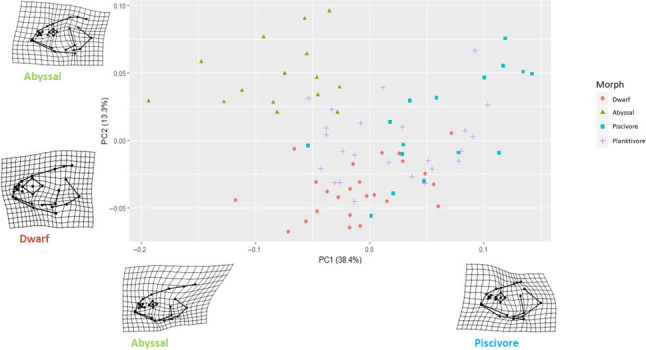
Principal component analysis of head shape illustrating extremes of head shape morphology in Arctic charr (red: Dwarf, green: Abyssal, blue: Piscivore, purple: Planktivore). The first two principal components are shown for the four morphs. Wireframe images illustrate head shape differences along the two first PC axes

To quantify differences in head shape among morphs, we retained the first six principal component axes (PC) explaining 78.1% of the variation in head shape across morphs. The first two PC separated three of the four morphs by head morphology, except the Planktivore morph, which overlapped with the Piscivore and Dwarf morphs (Figure 3). The first PC (38.4% of total variance) revealed two different head shapes corresponding to Abyssal and Piscivore morphs. The Piscivore had a larger head depth and a larger eye than the Abyssal. The second PC (13.3% variance) separated Abyssal from Dwarf, where the Abyssal morph had the smallest eyes and the Dwarf morph had relatively larger eye size than the other three morphs.

### 3.2 Morph prediction using random forest

**FIGURE 4:**
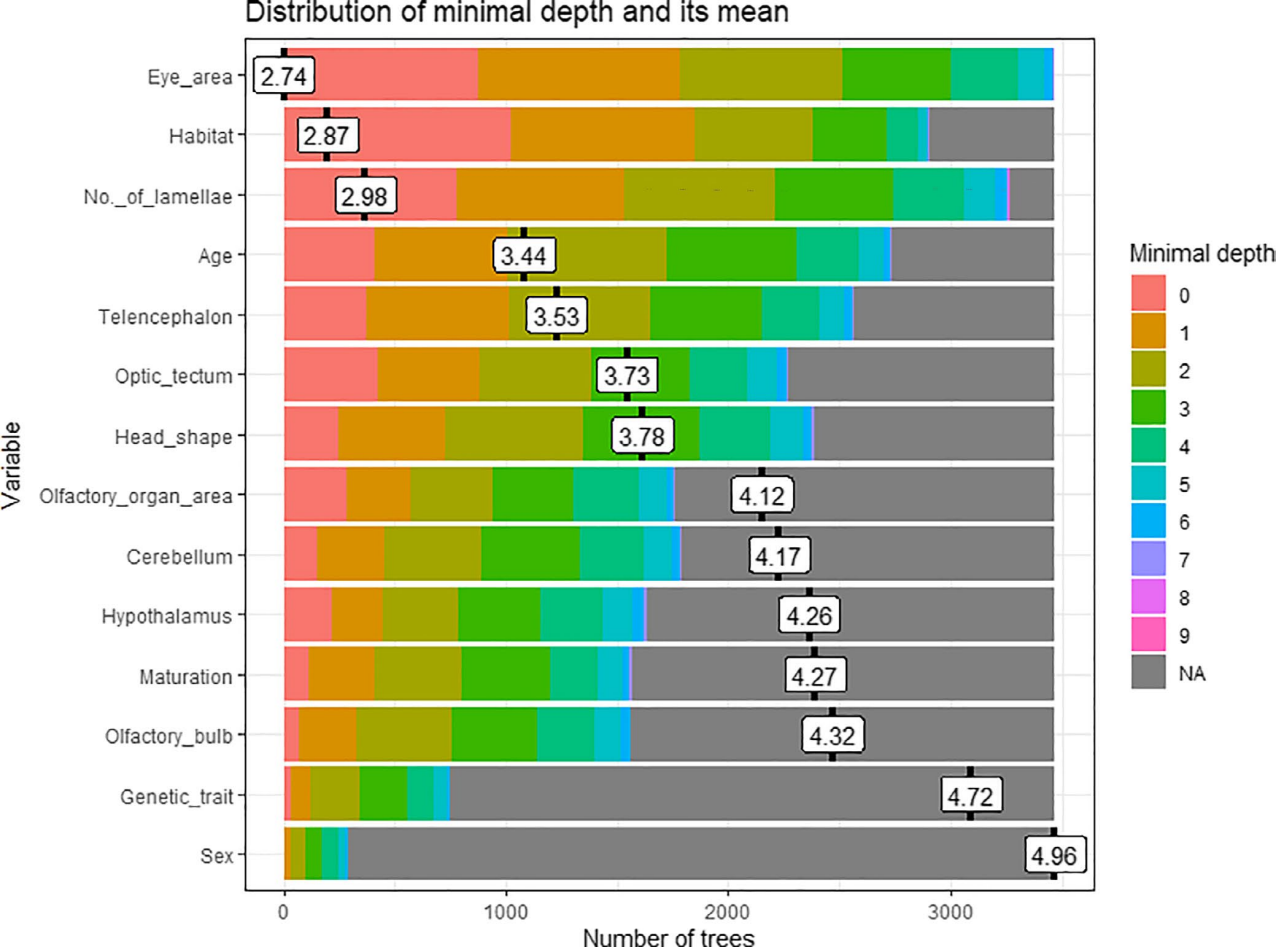
Variable importance based on minimum depth from the random forest analysis, which represents the consensus across trees (i.e., the higher the variables and the lower the depth on this figure, the more frequently and early the variable was selected to make the split, i.e., the more important the variable is). Results from the random forest analysis for the response variable morph. Note that we used the residuals of the measured variables obtained from the log‐log regressions to correct for size. Number of trees grown were set to 5,000. The importance of the variables is measured with the minimal depth (indicated with different colours inside the horizontal bar for each variable) and its mean (indicated in the white box). Minimal depth is the average distance between the root of a tree and the node/split where a given variable was used. Smaller values of the minimal depth indicate early contribution of the variable, that is, more discriminating power. NAs represent all variables not picked for a given split

For the morph classification, the prediction accuracy was 80%. The most important variables to predict the morph class were eye area, habitat, and number of lamellae (Figure 4). These variables were selected for early in the trees, which indicates that they have a great role in partitioning the data.

The variables from the ALE plots showed the effects and how they changed across the different classes (e.g., morphs).

**FIGURE 5:**
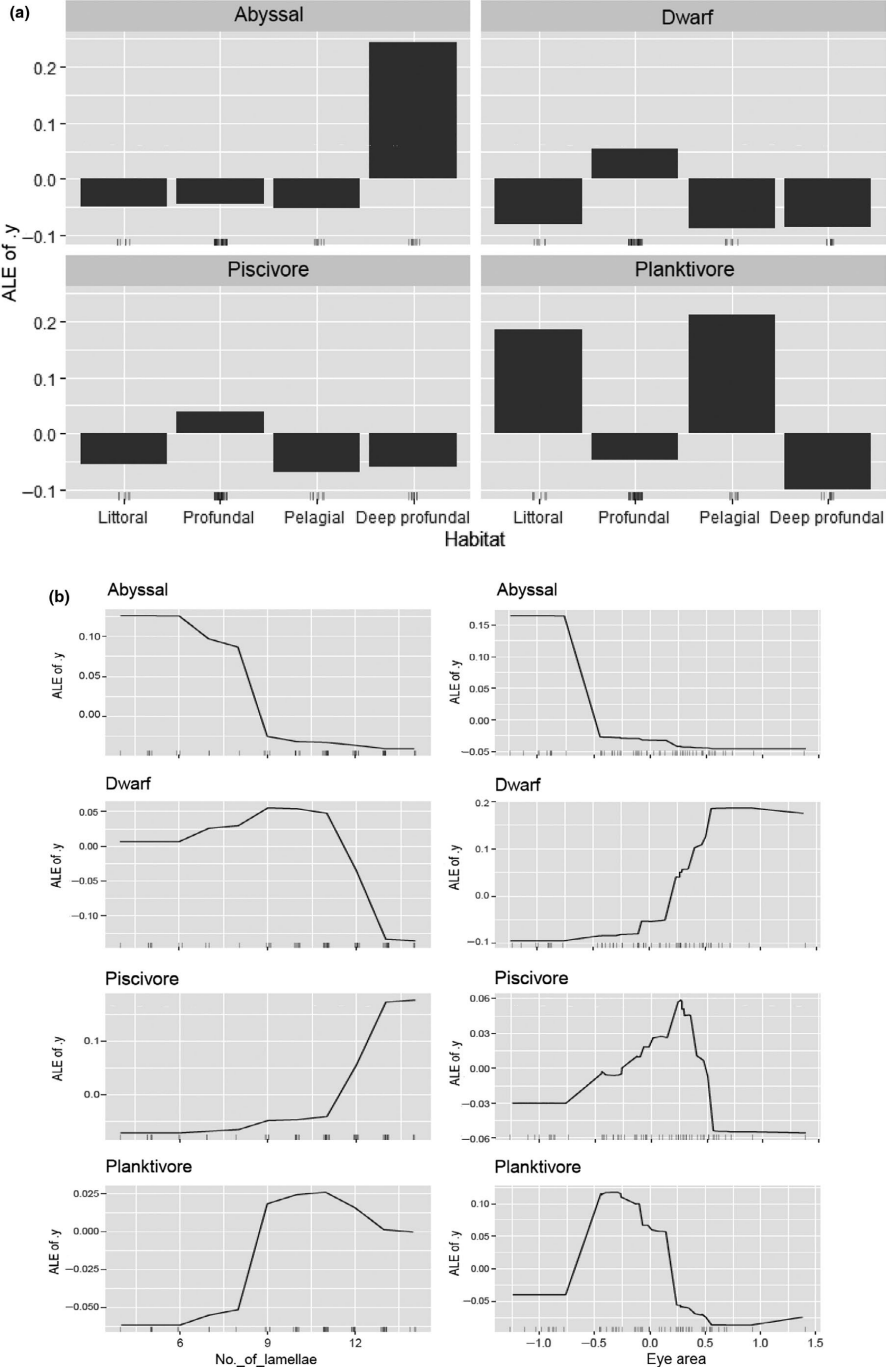
(a) Accumulated local effect (ALE) plots for habitat. Bars indicate the contribution of a given predictor, relative to the overall prediction of the model (at ALE of *y* = 0). Here, positive values of bars indicate higher prediction for a specific morph and negative values indicate lower effect on predicting morph (i.e., lower probability to be a determinate morph in that specific habitat). For instance, the probability of being Abyssal morph is higher in the deep‐profundal habitat. (b) Accumulated local effect plots for number of lamellae and eye area residuals,. ALE plots show the marginal effect of a variable on the predictions from the model. For instance, deep‐profundal habitat (a) and an eye area residuals smaller than −0.8 (b) have a high contribution on predicting the Abyssal morph. Lines indicate the contribution of these predictors, relative to the overall model prediction. The maximum values of line indicate highest prediction of given morph, for example, prediction of Dwarf morph is highest with eye area residuals larger than 0.5 and number of lamellae ranging from 9 to 11, whereas for Abyssal morph are <−0.8 and <6, respectively

For habitat, the fish caught in the deep‐profundal habitat were predicted to most likely be the Abyssal morph (Figure 5a). For the shallow‐moderate profundal habitat, the Dwarf or the Piscivore morphs were predicted. For the littoral and pelagial habitat, the Planktivore morph was predicted.

For the number of lamellae, the Abyssal morph was predicted to have less than 6 lamellae (Figure 5b, first panel). The Dwarf morph was predicted to have between 9 and 11 lamellae. The Piscivore morph was predicted to have more than 11 lamellae, and the Planktivore morph between 9 and 11.

For the eye area residuals, the Abyssal morph was predicted to have a value smaller than −0.8 (Figure 5b, second panel). The Dwarf morph was predicted to have the eye area residuals larger than 0.5. If the eye area residuals ranges from −0.3 to 0.3, the model predicted belonging to the Piscivore class. There is a higher prediction of being Planktivore morph when the eye area residuals were between −0.8 and −0.3.

### 3.3 Brain region and olfactory organ variation

The four morphs varied in brain region volumes. The largest absolute brain volumes were found in the Piscivore morph, followed by the Planktivore morph, whereas the Abyssal morph had the smallest (Table 1). The largest absolute brain region in all the morphs was the optic tectum, whereas the smallest was the olfactory bulb (Table 1). The optic tectum and the cerebellum were both larger in the Piscivore and the Planktivore morphs in comparison with the other two morphs (Table 1). The Abyssal morph had the smallest absolute optic tectum size compared with the other morphs. Within the Abyssal morph, the largest region was the optic tectum, followed by the cerebellum (Table 1). Comparing the Abyssal and the Piscivore morphs, the olfactory bulb represented a 12.8% and a 6.5% of the optic tectum in size, respectively (Table 1). In the case of the Dwarf and the Planktivore, the olfactory bulb represented a 7.2% and a 5.3% of the optic tectum in size, respectively. Therefore, there is an increase of the olfactory bulb in size in the Abyssal morph compared with the other three morphs.

**TABLE 1:** Summary of the Arctic charr measurements: number of individuals (*n*), sex (number of males/females), mean body length (±*SD*), mean of number of lamellae (±*SD*), mean of the absolute size of olfactory organ area (±*SD*), and mean of the absolute brain region volume (±*SD*) measured from four Arctic charr morph in Lake Tinnsjøen

Morph	*n*	Sex	Body length (mm)	Number of lamellae	Olfactory organ area (mm^2^)	Olfactory bulb (mm^3^)	Telencephalon (mm^3^)	Optic tectum (mm^3^)	Cerebellum (mm^3^)	Hypothalamus (mm^3^)
Abyssal	12	6/6	78.58 ± 10.66	5.67 ± 1.07	0.31 ± 0.12	0.10 ± 0.04	0.37 ± 0.16	0.78 ± 0.23	0.72 ± 0.30	0.17 ± 0.07
Dwarf	22	12/10	113.82 ± 21.50	10.45 ± 1.37	0.89 ± 0.27	0.24 ± 0.06	0.91 ± 0.37	3.32 ± 1.24	2.33 ± 1.00	0.48 ± 0.16
Piscivore	16	10/6	217.38 ± 92.96	12.75 ± 0.93	2.11 ± 1.30	0.74 ± 0.70	3.20 ± 3.07	11.31 ± 9.35	7.77 ± 6.96	1.28 ± 1.13
Planktivore	22	10/12	180.36 ± 67.42	11.41 ± 1.53	1.49 ± 0.64	0.54 ± 0.62	1.52 ± 1.10	10.14 ± 7.56	6.40 ± 4.90	0.86 ± 0.43

Results from the ANOVA revealed all traits were significantly different (*p* < .05), except genetic trait, sex, and olfactory bulb (Table 2). Eye area, habitat, and number of lamellae were the only variables that had significant differences across all morph comparisons, except in one comparison (Piscivore–Planktivore, Dwarf–Piscivore, and Dwarf–Planktivore, respectively), being the same variables selected as most important in the random forest. The Piscivore morph had the highest number of lamellae followed by the Planktivore morph, whereas the Abyssal morph had the lowest number of lamellae (Table 1). The Abyssal morph presented significant differences in the olfactory organ area, habitat, number of lamellae, optic tectum, hypothalamus, and eye area when it was compared with the other three morphs (Table 2). The most different morph was the Abyssal when compared with the Piscivore and Planktivore morphs (Table 2).

**TABLE 2:** Results from ANOVAs and post hoc Tukey's HSD tests indicating the difference of trait means and significant level tests between Arctic charr morphs

	ANOVA		Tukey's HSD tests					
	*F*_3,68_	*p*	Abyssal–Dwarf	Abyssal–Piscivore	Abyssal–Planktivore	Dwarf–Piscivore	Dwarf–Planktivore	Piscivore–Planktivore
Genetic trait	2.56	.06	−0.08	0.27	−0.12	0.35	−0.05	−0.39
Habitat	135.50	.00***	3.00***	3.00***	2.09***	−8.88e−16	−0.91***	−0.91***
Sex	0.37	.78	0.05	0.13	−0.05	0.08	−0.09	−0.17
No. of lamellae	75.15	.00***	4.79***	7.08***	5.74***	2.30***	0.95	−1.34*
Olfactory organ area	10.88	.00***	0.52***	0.43***	0.39***	−0.09	−0.13	−0.04
Olfactory bulb	1.65	.19	0.29	0.23	0.18	−0.06	−0.12	−0.05
Telencephalon	4.51	.01**	0.28	0.29	−0.06	0.00	−0.34*	−0.34*
Optic tectum	11.96	.00***	0.66***	0.49**	0.76***	−0.18	0.10	0.28
Cerebellum	4.63	.01**	0.43*	0.26	0.48**	−0.16	0.06	0.22
Hypothalamus	4.59	.01**	0.48**	0.37*	0.39*	−0.11	−0.09	0.02
Eye area	59.66	.00***	1.43***	1.01***	0.93***	−0.42***	−0.50***	−0.09
Age	9.56	.00***	0.88	2.21*	−1.03	1.33	−1.91**	−3.24***
Head size	4.64	.01**	301.10	811.66**	596.38*	510.56	295.28	−215.28
Maturation	4.39	.01**	0.27	0.13	−0.23	−0.15	−0.50**	−0.35

Note

We used the residuals of the measured variables obtained from the log‐log regressions to correct for size. Level of significance (*p*): *.01 < *p *≤ .05; **.001 < *p *≤ .01; ***p ≤ .001.

## 4 DISCUSSION

We found differences in the brain sizes among the four morphs of Arctic charr corresponding to their niche utilization. The optic tectum was the largest absolute brain region in the Piscivore and Planktivore morphs, which could be related to using a habitat with more light than the other two morphs. Comparing the olfactory bulb with the optic tectum in size, the olfactory bulb was larger in the Abyssal morph than in the other three morphs, suggesting that smell likely has a more relevant role than in the other morphs. The Piscivore morph presented the largest brain region volumes, whereas the Abyssal had the smallest, followed by the Dwarf morph. In the random forest analysis, eye area, habitat, and number of lamellae were the most important variables to classify the morphs suggesting differences in foraging and mating behaviour as well.

Based on the head morphology of Arctic charr, three of the four morphs were more distinguishable than the Planktivore morph (i.e., the most generalist morph).

### 4.1 Random forest analyses verify four morphs of Arctic charr

The deep‐profundal Abyssal morph presented the largest morphological differences compared with the other morphs, presenting a very distinct head shape and the smallest eyes and body length. The Dwarf and Piscivore morphs have evolved common head and body shapes, likely through parallel adaptation for occupying the shallow‐moderate profundal habitat, and both differ from the Planktivore morph, which has small eyes and head compared with the body size. Although both profundal morphs differ in head and body size (e.g., the Dwarf morph has smaller head, mouth, and body than the Piscivore morph), this is probably associated with diet preferences. From our results, it appears that there is certain selection pressure on vision or smell depending on the habitat and foraging behaviour. For instance, the morphs living in low light conditions could rely more on their vision developing larger eyes to detect their prey. Normally, the Piscivore morph of Arctic charr lives in the pelagic or littoral habitats (Adams et al., 1998; Power et al., 2005), but in Fennoscandia it seems that the piscivore morph mainly occupies the profundal habitat such as in Lake Tinnsjøen and Lake Skogsfjordvatn, Norway (Skoglund et al., 2015). It is likely that the Piscivore morph in Lake Tinnsjøen utilises several habitats in the lake, such as littoral, shallow‐moderate profundal, and deep‐profundal to seek for prey due to low density of fish (Østbye et al., 2020).

Both profundal morphs had larger eyes than the other morphs, but the Dwarf morph had the largest relative eye size. The larger eye size in the Dwarf morph may be favoured for feeding on small prey in habitats of low light conditions, whereas the eye size in the Piscivore could be due to feeding on more active prey, which can facilitate the detection of prey (Huber et al., 1997; Schliewen et al., 2001). The Piscivore also had a larger mouth, more robust head and larger body size than the other three morphs, which could be adaptations to enhance predation on other fish. Adams et al. (1998) also reported larger eye size in other piscivore morphs of Arctic charr likely related to predation behaviour, reaching a larger size and living longer than the other morphs. Morphological differences suggest different evolutionary pressures across and within habitats.

Our random forest analysis indicates that eye area, habitat, and number of lamellae seem to be good indicators for classifying morphs. These variables also showed differences among the morph comparisons in the ANOVA analyses, suggesting these predictors could have an important role in the morph diversity. The accuracy of the random forest was 80%. Here, having a larger dataset would most likely give a higher accuracy.

### 4.2 Habitat specialization and optic tectum volume

Living in a deepwater habitat means adaptation to the darkness, high pressure, low temperature, monotony, and a limitation in food resources (e.g., low prey densities). The limits of food abundance likely varies temporally and seasonally, affecting, for example, the fatty acid composition in the brain (Menzies, 1965; Patton, 1975; Roots, 1968). A reduction of vision can be a strategy to save energy in habitats with limited food and where vision can be not needed for feeding or predation avoidance (Moran, Softley, & Warrant, 2015). The Abyssal morph had the smallest eyes and optic tectum. The reduction in eye size across depth can indicate a decrease in the importance of vision due to a decrease in light irradiance (Huber et al., 1997). In addition, studies on cave and surface forms of the Mexican blind cavefish (*Astyanax mexicanus*) and medaka (*Ozyzias latipes*) showed that an increase in eye size, promoted by light irradiance, can affect the growth of the optic tectum (Ishikawa, Yoshimoto, Yamamoto, & Ito, 1999; Ishikawa et al., 2001; Soares, Yamamoto, Strickler, & Jeffery, 2004). Therefore, an increase of light would drive an increase in the size of the eye and optic tectum. In Lake Tinnsjøen, there is no light at 460 m, explaining the small eyes and small optic tectum size found in the Abyssal morph compared with the other three morphs, where vision can be more important. The presence of visual stimuli, such as bioluminescence, may determine the eye and optic tectum sizes in these kind of environments such as observed in the deep sea (Wagner, 2001).

The differences found in eye size and optic tectum, and even in the olfactory bulb, can be related to mating behaviour. Arctic charr has characteristically bright breeding coloration with a red belly and secondary sexual traits such as lower jaw type, which shows pronounced individual variation and potentially contribute to mate selection (Janhunen, Peuhkuri, Primmer, Kolari, & Piironen, 2011; Kekäläinen, Vallunen, Primmer, Rättyä, & Taskinen, 2009). A distinguished coloration may be important for female mate preferences in well‐illuminated habitats, where vision will be of higher importance than in dark habitats. In Lake Tinnsjøen, the pale coloration presented in the Abyssal morph most likely indicates a lesser importance of coloration in mating than in the other three morphs living in habitats with more light. Sensory‐driven divergence in visual capacities during speciation has been documented for cichlids as well, with a clear link to mate selection (Seehausen et al., 2008). However, we still have a very limited amount of studies of colour vision in Arctic charr morphs (Kahilainen et al., 2016). Major histocompatibility complex (MHC) genes can influence mating choice and kin recognition through olfaction, where females can reject mates with high differentiation in the MHC genotypes (Landry et al., 2001; Milinski et al., 2005; Olsén, Grahn, Lohm, & Langefors, 1998). Wild Arctic charr populations show differences in MHC genotypes within and among morphs, and diversity of polymorphisms in MHC can be linked to a lower amount of parasites (Conejeros et al., 2014; Eizaguirre & Lenz, 2010; Kekäläinen et al., 2009). Large variation in ecological niches and colouration of different Arctic charr morphs in Lake Tinnsjøen would provide a nice setting for parasite and MHC genotype studies as well as experimental tests for sexual selection potentially acting on phenotypes.

#### 4.2.1 Smell perception capacities

All morphs presented differences in the number of lamellae and the Abyssal morph showed differences in the olfactory organ area when compared with the other morphs. The Piscivore was the morph with the largest absolute size of the olfactory bulb, olfactory organ, and largest number of lamellae, followed by the Planktivore and the Dwarf morphs, whereas the Abyssal had the smallest. Wagner (2001) found that species relying more on visual foraging have larger optic tectum than species relying on the smell, which have a larger olfactory bulb. However, the combination of different stimuli and the occupation of different habitats may have determined the sensory preferences, developing specific brain regions independently.

Regarding olfactory lamellae, previous studies have found that the size of the olfactory organ and the number of lamellae increases with fish size (Atta, 2013; Halama, 1982; Kasumyan, 2004; Kudo, Shinto, Sakurai, & Kaeriyama, 2009). These findings were also corroborated by Olsén (1993), who founded that the size and number of lamellae increased with the body size of Arctic charr reared in the laboratory. Our study also supports these studies, presenting the largest number of lamellae and larger olfactory organ area in the largest morph (i.e., Piscivore morph) and the smallest in the minute morph (i.e., Abyssal morph).

#### 4.2.2 Brain region volumes differ among the morphs

In this study, the brain regions had different volumes among the morphs. The five small brain regions found in the Abyssal morph could be a response to low availability of energy through food (e.g., food quality/quantity), as has been found in a study of *Poecilia mexicana* that live in cave habitats and has a reduction of the optic tectum size and the total brain size (Eifert et al., 2015). A small brain can be a strategy to reduce energy expenditure in cave habitats (Tobler, 2008; Tobler et al., 2006); this can also apply to deep‐profundal habitat in Lake Tinnsjøen, where environmental parameters, such as light, temperature, and low resources, in many ways resemble a similar habitat to caves. Thus, is it the lack of light or food, or both that caused brain reduction in cave fish and in the deep‐profundal Abyssal morph? As the brain is an energetically expensive organ, a reduction of the relative brain size likely reflects a decrease in their metabolic rate, as seen in other species (Poulson, 1963, 2001; Shi et al., 2018). These small brain region sizes are probably due to a reduction in the physical space of the skull, constraining the brain size. Head morphology of the Abyssal morph and its cranial space may force some modifications on the structure of the brain regions due to spatial constrains (Striedter & Northcutt, 2006). Hypoxia can also be another factor that can reduce the brain size, as observed in other species (Chapman & Hulen, 2001). However, Lake Tinnsjøen is an oxygen‐rich deep‐water lake across the different habitats. Therefore, oxygen is not likely to be a factor constraining the brain size. Pressure might also have an effect on the brain size, especially in the deep habitat where the Abyssal morph lives. Thus, we have to consider different factors when it comes to brain morphology depending on the habitat where the morphs live.

According to the *mosaic evolution hypothesis*, each brain region is able to develop independently from the others (Hager, Lu, Rosen, & Williams, 2012; Liem, 1978). Our study supports this hypothesis, where the foraging behaviour and habitat specialisation of the different morphs most likely explain the variation in the brain regions we observed. Previous studies have found that, depending on environmental conditions, presence of conspecifics and ecological and behavioural conditions, there are certain brain regions that can be more important, and more developed than others (Gonda et al., 2009; Kihslinger, Lema, & Nevitt, 2006; Kihslinger & Nevitt, 2006; Kotrschal et al., 1998; Lisney et al., 2007). Thus, the pattern observed in brain region differentiation in the four Arctic charr morphs could be due to a rather complex set of putative explanations.

## 5 CONCLUSIONS

In summary, we found differences among morphs in body size, eye area, and number of lamellae, which were associated with habitats and diet used by morphs. For instance, large body size is attained from energy rich prey, that is, fish, in the case of the Piscivore morph or productive habitats in the Planktivore morph. It seems that living in different habitat conditions, such as lack of light and food limitation, affects brain morphology as showed in the small brain regions of the Abyssal morph. The optic tectum was the largest in the Piscivore and Planktivore morphs living in more illuminated habitats compared to the Abyssal, which had the smallest, suggesting a less developed vision. These clear relationships between brain traits and habitats suggest long‐term niche specialization, which may originate from phenotypic plasticity or adaptive evolution. These relationships warrant further empirical and experimental studies. As our study present the first brain region study from *Salvelinus*, there is need for studies in other polymorphic species, such as *Coregonus* and *Cottus*, to test the generality of our findings.

## CONFLICTS OF INTEREST

The authors declare no conflicts of interest.

## AUTHOR CONTRIBUTION

**Ana‐Maria Peris Tamayo:** Conceptualization (equal); Data curation (lead); Formal analysis (lead); Writing‐original draft (lead); Writing‐review & editing (lead). **Olivier Devineau:** Formal analysis (equal); Supervision (equal); Writing‐review & editing (equal). **Kim Præbel:** Conceptualization (lead); Data curation (equal); Funding acquisition (lead); Project administration (lead); Resources (lead); Supervision (equal); Writing‐review & editing (equal). **Kimmo K. Kahilainen:** Conceptualization (supporting); Formal analysis (supporting); Supervision (supporting); Writing‐review & editing (equal). **Kjartan Østbye:** Conceptualization (lead); Data curation (equal); Funding acquisition (lead); Project administration (lead); Resources (lead); Supervision (equal); Writing‐review & editing (equal).

## Data Availability

Data are available in the repository Dryad at: https://doi.org/10.5061/dryad.15dv41nvt.
